# Association between Appendectomy and Subsequent Colorectal Cancer Development: An Asian Population Study

**DOI:** 10.1371/journal.pone.0118411

**Published:** 2015-02-24

**Authors:** Shih-Chi Wu, William Tzu-Liang Chen, Chih-Hsin Muo, Tao-Wei Ke, Chu-Wen Fang, Fung-Chang Sung

**Affiliations:** 1 Trauma and Emergency Center, China Medical University Hospital, Taichung, Taiwan; 2 School of Medicine, China Medical University, Taichung, Taiwan; 3 Division of Colorectal Surgery, Department of Surgery, China Medical University Hospital, Taichung, Taiwan; 4 Management Office for Health Data, China Medical University and Hospital, Taichung, Taiwan; 5 Department of Surgery, Changhua Christian Hospital, Changhua, Taiwan; 6 Institute of Clinical Medical Science, China Medical University College of Medicine, Taichung, Taiwan; UMR INSERM U866, FRANCE

## Abstract

**Objectives:**

The appendix may modulate colon microbiota and bowel inflammation. We investigated whether appendectomy alters colorectal cancer risk.

**Methods:**

We identified a cohort of 75979 patients who underwent appendectomy between 1997 and 1999 based on the insurance claims of Taiwan. A comparison cohort of 303640 persons without appendectomy was selected randomly, frequency matched by age, sex, comorbidity and entry year was also selected. We monitored subsequent colorectal cancer development in both cohorts.

**Results:**

The overall colorectal cancer incidence was 14% higher in the appendectomy patients than in the comparison cohort (p <0.05): the highest incidence was observed for rectal cancer, and the lowest incidence was observed for cancer of the cecum-ascending colon for both cohorts. Men were at higher risk than women. Subjects ≥ 60 years had an HR of 12.8 compared to those <60 years. The incidence of colorectal cancer was much higher in 1.5-3.5 years post appendectomy follow-up than for the comparisons (HR of 2.13). Patients who received an incidental appendectomy had an HR of 2.90 when compared to the comparisons.

**Conclusions:**

Results of our study suggest that appendectomy in patients with appendicitis is likely associated with the development of colorectal cancer in the post-surgery period.

## Introduction

Colorectal cancer is a common cancer that results in more than 600,000 deaths globally each year.[1] The risk factors of colorectal cancer include old age, male gender, a low-fiber diet, smoking, drinking, diabetes, genetics and environment.[2] Inflammation has also been implicated in the risk of cancers.[3] Chronic intestinal inflammation, namely inflammatory bowel disease (IBD), is recognized as an important risk factor that promotes the development of colon cancer,[4–5] although the cellular and microbial mechanisms remain unclear. Recent studies have found that up-regulated Interleukin-17, Interleukin-23 and signal transducer and activator of transcription (STAT 3) play an important role in driving tumor growth in patients with intestinal inflammation.[5–7]

The human gut microbiota plays a critical role in both luminal diseases and systemic diseases (e.g., diabetes mellitus) of the host.[2] Inflammation may interact with the changes in gut microbiota and development of colorectal cancer.[1, 8–9]

The vermiform appendix in humans is generally regarded as a vestigial structure. However, studies suggest that it serves as a “safe house” for biofilm formation to preserve and protect commensal bacteria needed for the epithelial mucosa in the colon.[10–11] Biofilms are most abundant in the appendix, cecum and ascending colon (right side colon). The microbiota and biofilm in the large bowel might be changed after removal of the appendix (i.e., appendectomy). The role of appendectomy in inflammatory bowel disease is of concern, whether as a cause or a consequence. Patients who underwent appendectomy are associated with a 1.6- to 2.1-fold increased risk of Crohn’s disease but are less likely to have ulcerative colitis.[12–15] The inflammatory responses differ in ulcerative colitis and appendicitis post-appendectomy[14–15]. These conditions reflect an essential role of the appendix in the ecology of the microbiota and inflammation of the large bowel. Studies have rarely investigated whether the removal of the appendix changes the microbiota ecology and risk of colorectal cancer. In the present study, using longitudinal insurance claims data, we explored the link between patients who underwent appendectomy and the risk of subsequent colon cancer.

## Materials and Methods

### Data source

The Taiwan National Health Insurance (NHI) Program is a single-payer system with approximately 99.5% of the population in Taiwan being covered. The Bureau of Health Insurance has entrusted the National Health Research Institutes to manage and establish the National Health Insurance Research Database (NHIRD) for researchers. We obtained claims data on inpatients from the catastrophic illness patient registry (CIPR) of the institutes. Patients with colorectal cancer applied for the CIPR cards based on colorectal pathology. The cardholders are exempted from the cost sharing required under the NHI program. Data files were linked by patient identifications, which had been scrambled before releasing the data to researchers according to the Personal Information Protection Act of the Department of Health. The International Classification of Diseases, 9th Revision. Clinical Modification ICD-9-CM was used to identify diseases. We conducted this study with the approval of the Ethics Review Committee at Chinese Medical University and Hospital.

Although written informed consent was not provided by the participants for the use of their clinical records in this study, the patient records/information was anonymized and de-identified prior to the analysis.

### Study subjects

Based on the inpatient claims data, we identified 84408 patients who underwent appendectomy (treatment codes of 47.0 and 47.1) due to appendicitis in 1997–1999. We excluded those with cancer history [(ICD-9-CM) 140–208] in CIPR (n = 1174), inflammatory bowel disease [IBD, (ICD-9-CM) 555 and 556] at admission before the operation date (at least 12 months from the start of 1996 to the operation date, n = 179), and patients with a diagnosis of CRC within 18 months of appendectomy (n = 7076).

**Fig 1:**
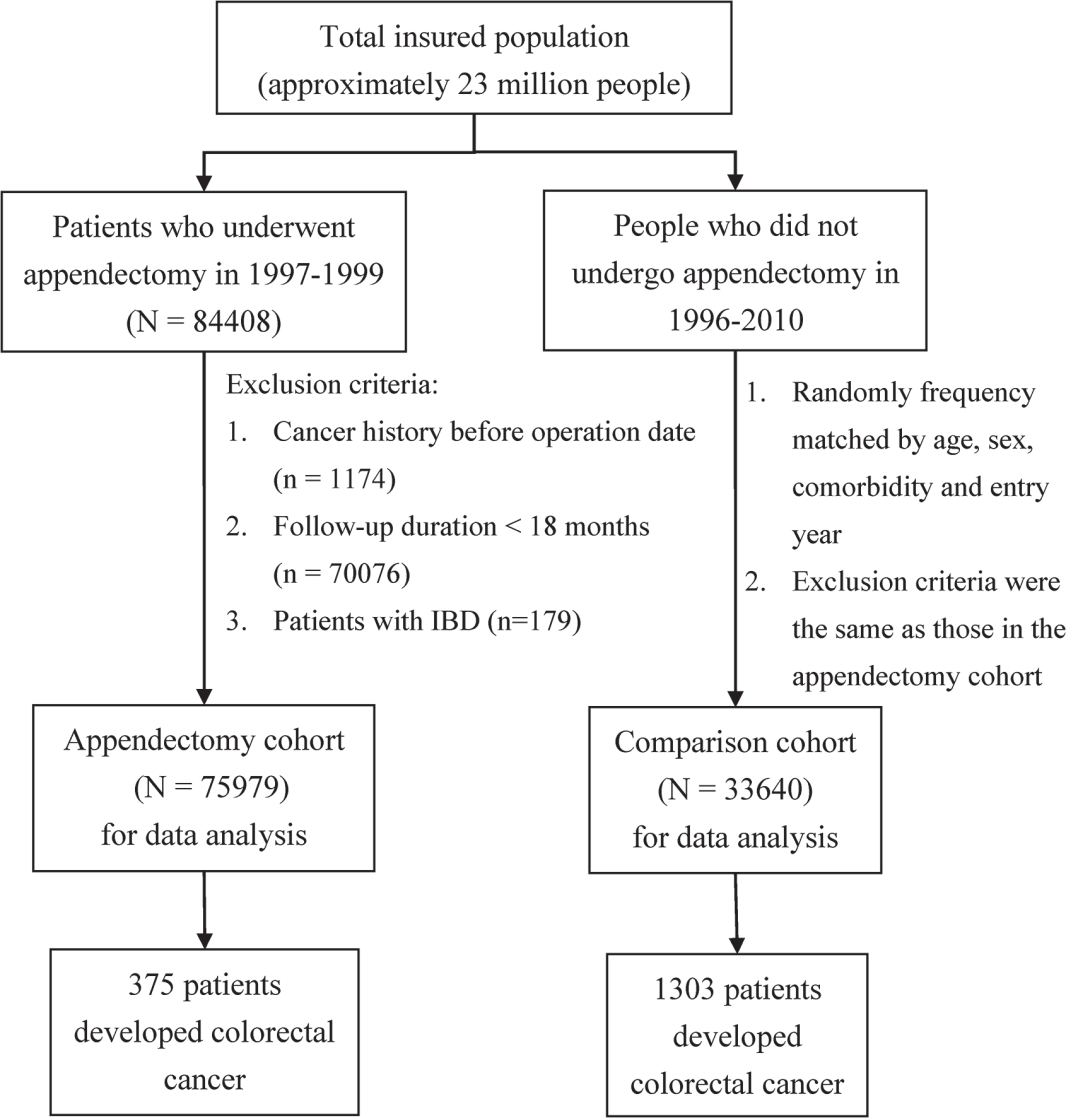
Flow chart for the study subjects.

The remaining 75979 patients were included in this study as the appendectomy cohort; the date of 18 months post appendectomy was defined as the entry date to initiate follow-up. Therefore, subjects in the appendectomy cohort had the washout period for cancer for a minimum of 30 months. For each appendectomy case, we randomly selected 4 comparison subjects from the inpatient claims data who were free of appendicitis and had no cancer history or IBD. They were frequency matched for age (5-year stratum), gender, and comorbidities including hypertension (ICD-9-CM 401–405), diabetes (ICD-9-CM 250), hyperlipidemia (ICD-9-CM 272) and colorectal adenomas (ICD-9-CM 211.3 and 211.4), and urbanization level of living areas (Fig. 1). All comorbidities were determined based on the hospitalization records by the entry date. The urbanization level was grouped into 5 levels according to NHI report: level 1 was the highest urbanization for living areas and level 5 was the lowest.

### End point

Study subjects were followed beginning on the entry date and ending on the date of colorectal cancer diagnosis (ICD-9-CM 153–154) identified in the CIPR. Alternatively, subjects were censored because of withdrawal from insurance, death or to the end of 2011.

### Statistical analysis

The data analysis compared the distributions of sex, age and comorbidities between the appendectomy and comparison cohorts. The standardized difference was used to quantify differences in means or prevalence between the two cohorts for continuous or categorical matching variables, respectively. The incidences of colorectal cancer were calculated for both cohorts by sex and age. Sex- and age-specific hazard ratios (HR) and 95% confidence intervals (CIs) of colorectal cancer for the appendectomy cohort compared with the comparison cohort were measured using Cox proportional hazard regression. Kaplan-Meier analysis was used to evaluate the cumulative incidence in the follow-up period between the two cohorts, examined using the log-rank test. We also estimated the site-specific colorectal cancer incidence incorporating HR estimates for the sites of hepatic flexure and transverse colon (ICD-9-CM 153.0 and 153.1), splenic flexure and descending colon (ICD-9-CM 153.2 and 153.7), sigmoid (ICD-9-CM 153.3), cecum and ascending colon (ICD-9-CM 153.4 and 153.6), rectal cancer (ICD-9-CM 154), mixed type and others. The effect of different types of appendectomy was also assessed by comparisons with the comparison cohort. In a future analysis, we will estimate the risk of colorectal cancer stratified by the follow-up years. All tests were performed using SAS version 9.3 (SAS Institute, Cary, NC, USA.) and R 2.11.1 (R Foundation for Statistical Computing, Vienna, Austria). A statistically significant level was set at p <0.05 with 2-sided probability.

## Results

We included 75979 patients in the appendectomy cohort and 303640 subjects who did not undergo appendectomy in the comparison cohort (Table 1). Both cohorts had a similar mean age (31.8 years), were predominantly composed of males and had similar prevalence of hypertension, diabetes, hyperlipidemia and colorectal adenomas.

**Table 1:** Distribution of demographic characteristics and comorbidity between the appendectomy and comparison cohorts.

	Comparison cohort N = 303640		Appendectomy cohort N = 75979		
	n	%	n	%	Standardized difference
Age, years					
< 20	97364	32.1	24349	32.1	0.00
20–39	117767	38.8	29457	38.8	0.00
40–59	59142	19.5	14802	19.5	0.00
60–79	26834	8.84	6731	8.86	0.0001
≥ 80	2533	0.83	640	0.84	0.0001
Mean (SD)†	31.8	(18.4)	31.8	(18.3)	0.002
Men	157835	52.0	38482	52.0	0.00
Urbanization					
1 (highest)	85507	28.2	21394	28.2	0.00
2	93712	30.9	23443	30.9	0.00
3	53977	17.8	13504	17.8	0.00
4	41513	13.7	10395	13.7	0.00
5 (lowest)	28931	9.53	7243	9.53	0.00
Comorbidity					
Hypertension	8872	2.92	2243	2.95	0.002
Diabetes	7160	2.36	1816	2.39	0.002
Hyperlipidemia	2467	0.81	630	0.83	0.002
Colorectal adenomas	633	0.21	217	0.29	0.02

**Fig 2:**
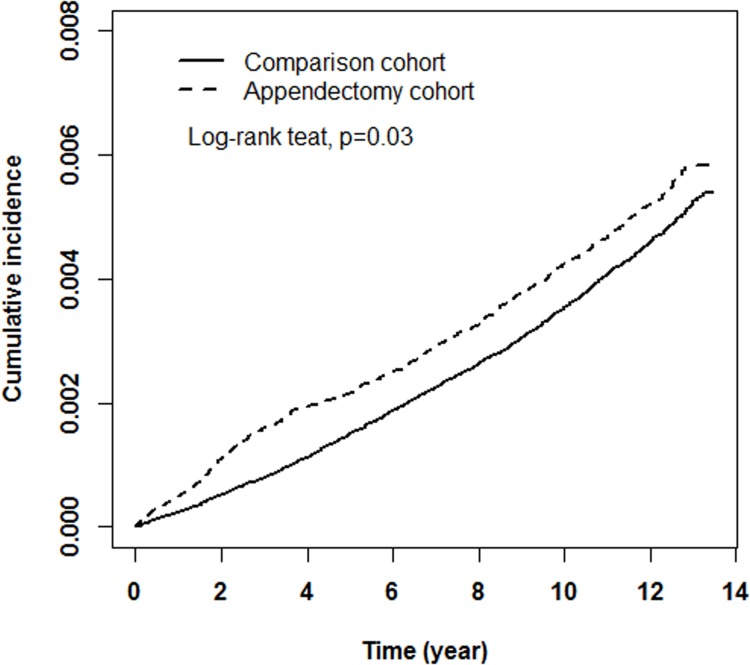
Cumulative proportional incidence of colorectal cancer between the appendectomy and comparison cohorts.

By the end of 2011, the incidences of colorectal cancer were 4.35 and 3.81 per 1000 person-years in cohorts that underwent and did not undergo appendectomy, with an HR of 1.14 (95% CI = 1.02–1.28) for patients with appendectomy compared to the comparisons (Table 2). Men had a higher incidence of the disease than women in both cohorts, with an HR of 1.15 (95% CI = 1.05–1.27). Patients who underwent appendectomy at age ≥ 60 years had a much higher incidence of colorectal cancer than the younger subjects in both cohorts; the patients 60 years and older had an HR of 12.8 compared with patients younger than 60 years. The age patterns in men and women were similar. The cumulative incidence for colorectal cancer in patients with appendectomy was 0.04% greater than the comparisons after the 14-year follow-up period (15.5-year post appendectomy follow-up) (Fig. 2).

**Table 2:** Incidence and hazard ratios of colorectal cancer by age and gender.

	Appendectomy Cohort		Comparison Cohort		Sex and age-specific	Between sexes and ages
	Events	Incidence†	Events	Incidence†	HR (95% CI)	HR (95% CI)
Overall	375	4.35	1303	3.81	1.14 (1.02–1.28)*	
Men	215	4.87	704	4.01	1.21 (1.04–1.41)*	1.15 (1.05–1.27)**
Women	160	3.81	599	3.60	1.06 (0.89–1.26)	1.00
Age, years						
< 60	172	2.16	662	2.10	1.03 (0.87–1.22)	1.00
≥ 60	203	30.46	641	24.59	1.24 (1.06–1.45)**	12.8 (11.6–14.1)***
Gender						
Men						
< 60 years	96	2.35	360	2.22	1.06 (0.85–1.33)	1.00
≥ 60 years	119	34.92	344	25.69	1.36 (1.10–1.67)**	12.9 (11.3–14.7)***
Women						
< 60 years	76	1.96	302	1.96	1.00 (0.78–1.28)	1.00
≥ 60 years	84	25.69	297	23.43	1.10 (0.86–1.40)	12.7 (11.0–14.7)***

† per 10000 person-years

* p<0.05

*** p<0.0001

Entry date: the date at the 18 months in the beginnings for appendectomy.

In general, the site-specific incidences of colorectal cancer were higher in the appendectomy cohort than in the comparison cohort (Table 3). The incidence of rectal cancer was the highest in both cohorts (1.85 vs. 1.67 per 10000 person-years). Cecum and ascending colon cancer had the lowest incidence in both cohorts. The incidence of colorectal cancer was 1.02- and 2.90-fold higher in patients who underwent an appendectomy and incidental appendectomy than in the comparisons (3.89 and 11.04 vs. 3.81 per 10000 person-years) (Table 4).

**Table 3:** Incidence of colorectal cancer by site and hazard ratio of the site-specific appendectomy cohort to the comparison cohort.

	Appendectomy cohort		Comparison cohort		
Location	Event, n	Incidence†	Event, n	Incidence†	HR (95% CI)
HF-Transverse	24	0.28	66	0.19	1.44 (0.90–2.30)
SF-Descending	11	0.13	48	0.14	0.91 (0.47–1.75)
Sigmoid	48	0.56	180	0.53	1.06 (0.77–1.45)
Cecum-Ascending	7	0.08	23	0.07	1.21 (0.52–2.82)
Others (153.8, 153.9)	63	0.73	202	0.59	1.24 (0.93–1.64)
Rectal cancer (154)	159	1.85	571	1.67	1.11 (0.93–1.32)
Mix	63	0.73	213	0.62	1.17 (0.89–1.55)

† per 10000 person-years

HF, hepatic flexure; SF, splenic flexure; Others (ICD-9 codes 153.8, 153.9)

Entry date: the date at the 18 months in the beginnings for appendectomy.

**Table 4:** Incidence and hazard ratio of colorectal cancer in different types of appendectomy.

Variable	N	Event no.	Incidence†	HR (95% CI)
Comparison cohort	303640	1303	3.81	1.00
Appendectomy cohort				
Appendectomy	71013	314	3.89	1.02 (0.90–1.16)
Incidental appendectomy	4966	61	11.04	2.90 (2.24–3.75)***

† per 10000 person-years

*** p<0.0001

Entry date: the date at the 18 months in the beginnings for appendectomy.

In the analysis stratified by follow-up the year, colorectal cancer appeared much earlier in the appendectomy cohort than in the comparison cohort, with incidences of 5.52 and 2.60 per 10000 person-years in 1.5–3.5 years post appendectomy follow-up, respectively, and an HR of 2.13 (95% CI = 1.63–2.77) for the appendectomy cohort. The incidence in the appendectomy cohort was declining, while that in the comparison cohort was not. The incidence in year 6.5 was slightly lower in the appendectomy cohort, with an insignificant HR of 0.98. (Table 5)

**Table 5:** Incidence and hazard ratio of colorectal cancer during post appendectomy follow-up.

Variable	Appendectomy cohort		Comparison cohort		
	Event no.	Incidence†	Event no.	Incidence†	HR (95% CI)
Post appendectomy follow-up years					
1.5–6.5	162	4.36	444	3.00	1.45 (1.21–1.74)***
> 6.5	213	4.34	859	4.43	0.98 (0.84–1.14)
Post appendectomy follow-up < 6.5 years					
1.5–3.5	83	5.52	156	2.60	2.13 (1.63–2.77)***
3.5–6.5	79	3.58	288	3.28	1.09 (0.85–1.40)

† per 10000 person-years

* p<0.05

** p<0.01

*** p<0.0001

## Discussion

In this large retrospective cohort study with a 14-year follow-up, we found that patients who underwent an appendectomy had a 1.14-fold higher risk of colorectal cancer than the general population. The risk of cancer was 12.8-fold higher for older subjects compared with younger subjects. The results are reliable because the robustness of this relationship is supported by accurate diagnosis of both appendectomy and colorectal cancer with histological confirmation.

Colorectal cancer is a common malignancy and is one of the major causes of cancer-related death in the world. Approximately 20% of all cases of the disease can be attributed to genetic factors.[16–17] Another large portion of the cases might be related to environmental causes rather than genetic factors. Among the environmental factors, the role of chronic intestinal inflammation in tumor development has received great attention in recent decade, particularly the roles of Crohn's disease and ulcerative colitis.[18–21] The large bowel microbiota plays a role in the development of colitis-associated cancer (CAC).[22–23] Ulcerative colitis has accounted for up to 18–20% of CAC, while Crohn's disease accounted for up to 8%.[20, 24–25] There are overlapping mechanisms in tumor formation between colorectal cancer and CAC.[26–28] On the other hand, previous studies on the relationship between appendix inflammation and colorectal cancer have been insufficient.[29–30]

A previous study reported that the number of natural killer cells in the appendix tissue is the highest in patients with appendix inflammation and lowest in patients with colon cancer and normal appendices.[29] Lai et al. used hospital medical records to evaluate 1873 patients with appendicitis and found that 16 of them also had colorectal cancer. No further follow-up observations were conducted. However, in their study, the colorectal cancer rate in appendicitis patients (8.54 per 1,000) is much greater than the incidence in the general population (3.19 per 10,000).[30] Appendicitis is thus considered the first manifestation of colon cancer, indicating a close relationship between the appendix and colon cancer. Our follow-up study also found that the colorectal cancer risk is higher for appendectomy patients than for the general population, although it is not as high as the rate found by Lai et al. The mechanism of cancer development associated with appendicitis may not be similar to the mechanism of cancer development associated with appendectomy.

This study also found that the cancer risk is higher for elderly patients who underwent appendectomy, particularly in older men. The higher incidences in older patients might be attributed to age-related decreases in immunity [31–33] and age-related changes in the large bowel microbiota, [34–36] which make the individual more susceptible to the development of malignancy.

The biofilms of the large bowel are abundant in the appendix, cecum and right side colon.[10] Impairing the growth of these biofilms may lead to dysbiosis and may make the tissue vulnerable to inflammation, ultimately leading to the development of colorectal cancer. [37] In the present study, the incidences of cancer among all colon sites are elevated after appendectomy. However, we found the lowest incidence of colorectal cancer in the cecum and the ascending colon in both cohorts (Table 3). The previous abundant biofilm in cecum and ascending colon may be with lesser degree of dysbiosis and lesser declination in biofilm protective effect than in the rectum, which resulted in lower potential for malignancy. This finding may support the “safe house” hypothesis of biofilms in the appendix.[10–11] Further study is needed to explore whether the protective effect of biofilms decreases after appendectomy.

It is important to note that the colorectal cancer incidence is higher within 3.5 years of follow-up for those with appendectomy. It is not clear whether the cancer risk in this period is associated with appendicitis or is associated with the impaired colon microbiota because of appendectomy. The risk decreases to the level of the general population 6.5 years later. The change in the colon microbiota after appendectomy may have only a short-term rather than long-term impact on colorectal carcinogenesis. The intestinal ecology may achieve balance as time passes. However, further investigation is required.

More than 500 widely diverse bacterial species of normal human commensal microbiota have been estimated to live in the gut. [38–39] Some specific strains of bacteria are pathogenic and can cause cancer.[40–43] Among the anaerobes, *Fusobacterium* species may link appendicitis and subsequent colorectal cancer.[44–46] *Fusobacterium nucleatum/necrophorum* are responsible for the majority of cases of acute appendicitis,[47–48] while *Fusobacterium* genomic sequences have been associated with IBD, including ulcerative colitis, and are enriched in colorectal carcinomas.[49–56] McCoy et al. found that subjects with a high abundance of *Fusobacterium* had an odds ratio of 3.66 (95% CI 1.37–9.74) to develop colorectal cancer [54]. It is plausible that patients undergoing appendectomy are at a higher risk of developing IBD and subsequent colorectal carcinogenesis because of lacking the function of appendix as”safe house”. [10–11, 53–56]

The older subjects had an HR of 12.8 compared with the younger subjects (Table 2). To the best of our knowledge, no report has emphasized age difference in the relationship between appendicitis and the development of colorectal cancer. In the present study, the distributions of baseline comorbidities were not significantly different between the appendectomy and comparison cohorts (Table 1). The increased hazard ratios in older subjects may be attributed to the alteration of the intestinal ecology after removal of the appendix rather than the impact of aging itself.

This study also showed that patients who undergo incidental appendectomy are at a higher risk of subsequent colorectal cancer (HR 2.90, Table 4). It is possible that such patients could in fact have undetected cancer or have related high-risk conditions. To reduce misclassification, we excluded patients with colorectal cancer that was detected within 18 months after the appendectomy. Therefore, removal of an ordinary appendix is also associated with a higher incidence of subsequent colorectal cancer. This result may provide further evidences in the “safe house” hypothesis of the appendix.

The association between appendectomy and subsequent colorectal cancer has been observed in several earlier studies carried out in the 1960s through the 1980s. [57–63] No study has ever longitudinally examined the development of colorectal cancer after appendectomy. The present large cohort study revealed that cancer incidence is higher in patients with appendectomy than in the comparisons for each colorectal site. The typical pathophysiology of colon cancer development has a typical time frame of near 10 years, with the polyp-to-cancer transformation cycle averaging 7–10 years.[64, 65] However, in our study, the overall colorectal cancer incidence in the 1.5–3.5 years of follow-up is much higher than that in later years. The finding that the incidence is consistently higher in later years may indicate that the risk remains without the protective role of appendix. This result may deserve attention and further studies.

Dysplastic adenomas are the most common form of premalignant precursor lesions [66]. In molecular features, the APC gene mutations present in the early phase of colorectal cancer formation with approximately 70% of colorectal adenoma.[67] Furthermore, activating mutations of the KRAS oncogene and inactivating mutations of the TP53 tumor suppressor gene could promote adenoma–carcinoma sequence.[68] However, in the present study, whether the shorter latent period for colorectal cancer after appendectomy is attributable to molecular changes after removing appendix is unclear. There may be genetic or environmental dispositions in the Taiwanese species, which deserves further study as well.

### Limitation of the study

This study has strengths of including a large study population, the longitudinal design, the reliable diagnosis and the high follow-up rate. However, certain limitations also exist. First, complete lifestyle information with respect to drinking, smoking, diet, socioeconomic status, and genetic factors was not available for the colorectal cancer risk adjustment. However, these factors are related to both appendectomy and colorectal cancer. Thus, the bias derived from this source is modest. Second, despite our meticulous study design and controlling for confounding factors, bias resulting from the retrospective nature of the study may have influenced our results. Third, all data used were anonymous. Therefore, relevant clinical variables, such as pathology findings, cancer staging, imaging results, and serum laboratory data were unavailable in our study. However, the data regarding appendectomy and cancer diagnoses were highly reliable.

### Conclusions

In conclusion, this follow-up study using large Asian population data shows that patients with appendicitis are at an elevated risk of colorectal cancer after appendectomy. The overall risk elevation is estimated for 14%, but much greater for the elderly patients. Although further study is necessary to determine whether appendicitis truly increases the cancer risk, and the mechanism by which the cancer risk increase might occur.
